# Patterns of vitamin D deficiency and functional resistance in cancer: a brief observational report

**DOI:** 10.3389/fmolb.2026.1790468

**Published:** 2026-05-26

**Authors:** Pradeep M. K. Nair, Shanmugam Sudarshan, Sekar Sivaranjani, Elangovan Karthika, Maruthanayagam Saranya, Ayswarya Rohini Pandian, Sridhar Shubakarini, Manickam Mahalingam, Jagadheesan Sugashwaran, Renu Mahtani, Girishankara K. S. Moodala, Karishma Silwal

**Affiliations:** 1 Mirakle Integrated Health Centre, Pollachi, India; 2 Dr. Renu Mahtani’s Autoimmunity Treatment Centre, Pune, India; 3 S-VYASA School of Yoga and Naturopathic Medicine, SVYASA University, Bangalore, India; 4 Institute of Advanced Bioscience, Keio University, Tsuruoka, Japan

**Keywords:** cancer metabolism, neoplasms, parathyroid hormone, vitamin D deficiency, vitamin D resistance

## Abstract

**Background:**

Although vitamin D deficiency is common in cancer populations, responses to vitamin D supplementation remain inconsistent. This suggests that circulating vitamin D levels may not fully capture its biological activity and that impaired vitamin D responsiveness, or functional vitamin D resistance, may be present in a subset of patients.

**Methods:**

A retrospective observational analysis of 220 patients with solid and hematological malignancies evaluated at an integrative oncology center in India. Serum 25-hydroxyvitamin D [25(OH)D] and intact PTH (iPTH) levels were assessed. Vitamin D deficiency (<20 ng/mL), insufficiency (20–29 ng/mL), and sufficiency (≥30 ng/mL) were defined using standard thresholds. Functional vitamin D resistance was defined as elevated iPTH (>65 pg/mL) in the presence of sufficient vitamin D. Distribution patterns were examined across cancer types, disease stage, metastatic status, and metabolic comorbidities.

**Results:**

Among 220 patients (mean age 57.6 ± 14.8 years), mean serum 25(OH)D was 22.2 ± 16.0 ng/mL, indicating a high prevalence of insufficiency. Serum 25(OH)D was inversely correlated with iPTH (p = 0.005). Advanced tumor stage was independently associated with vitamin D deficiency, while dyslipidemia was paradoxically associated with lower odds of deficiency. Functional vitamin D resistance was identified in 8 of 206 evaluable patients (3.9%) and was significantly associated with type 2 diabetes mellitus and dyslipidemia, but not with tumor stage or metastatic status.

**Conclusion:**

Vitamin D deficiency and insufficiency are common in cancer, whereas functional vitamin D resistance represents a distinct and less frequent phenotype associated with metabolic comorbidities. Assessment of iPTH alongside serum vitamin D may provide additional insight into vitamin D endocrine responsiveness in oncology settings.

## Introduction

Vitamin D plays a central role in calcium–phosphate homeostasis, immune modulation, mitochondrial function, and cellular differentiation. In cancer, low serum vitamin D levels have been associated with adverse outcomes, including disease progression, fatigue, sarcopenia, and reduced survival. (Jeon and Shin, 2018; Feldman et al., 2014). A growing body of epidemiological and clinical evidence links low serum 25-hydroxyvitamin D [25(OH)D] levels to increased cancer incidence, advanced disease stage, cancer-related fatigue, sarcopenia, treatment intolerance, and reduced overall survival across multiple malignancies, including breast, colorectal, prostate, and hematological cancers. (Feldman et al., 2014; Zhang et al., 2024).

Consequently, routine screening and supplementation of vitamin D have become integral components of supportive and integrative cancer care. Further, vitamin D deficiency (or insufficiency) is one of the most common causes of elevated PTH. (Silverberg, 2007). However, vitamin D status and PTH do not always follow a predictable feedback relationship. In some individuals, PTH remains inappropriately elevated despite apparently sufficient 25(OH)D levels, a phenomenon described as acquired “vitamin D resistance” or reduced biological responsiveness. (Lemke et al., 2021). This phenomenon has been well described in conditions such as chronic kidney disease, obesity, aging, and chronic inflammatory disorders, where systemic inflammation and metabolic dysregulation may impair vitamin D receptor (VDR) signaling. (Lemke et al., 2021).

Altered vitamin D metabolism within tumor and immune cells, including reduced conversion to the active form 1,25-dihydroxyvitamin D or increased catabolism via CYP24A1 overexpression, may contribute to functional deficiency despite adequate circulating stores. (Larriba and Muñoz, 2010). Persistently elevated PTH in this context may itself exert deleterious effects, promoting bone resorption, muscle catabolism, fatigue, and metabolic instability, features that are commonly observed in advanced cancer and cancer cachexia. (Fraser, 2009). Several randomized trials and observational studies also report that correction of serum 25(OH)D does not uniformly translate into improvements in clinical outcomes, or survival. (Zhang et al., 2022). This suggests that circulating vitamin D concentrations alone may be insufficient to reflect its true biological activity.

In summary, both vitamin D deficiency and a potential impairment of vitamin D action are relevant in cancer care. Yet most studies in cancer patients have focused only on deficiency. To our knowledge, the frequency and clinical correlates of functional vitamin D resistance (elevated PTH despite normal 25(OH)D) in a cancer cohort have not been evaluated. We sought to determine how common vitamin D deficiency, insufficiency, and functional resistance are. Rather than testing a definitive causal hypothesis, this analysis was designed to describe trends and generate hypotheses regarding potential functional heterogeneity in vitamin D responsiveness among cancer patients.

## Materials and methods

### Study design and setting

This retrospective observational study was conducted at an integrative oncology center in India. Clinical, demographic, and biochemical data were retrospectively extracted from patient medical records of individuals evaluated as part of routine oncological and metabolic assessments. The study was approved by the institutional ethics committee of the integrative oncology center. As this was a retrospective analysis of anonymized clinical data, the requirement for informed consent was waived.

### Study population

Adult patients (≥18 years) who visited the facility between January 2024 and December 2025, with a confirmed diagnosis of cancer, who had available measurements of serum 25-hydroxyvitamin D [25(OH)D] and intact parathyroid hormone (iPTH) were eligible for inclusion. Patients were included irrespective of their cancer type, stage, or treatment modality. No exclusion criteria were applied to preserve real-world clinical heterogeneity. As this study was a retrospective exploratory analysis, no *a priori* sample size calculation was performed. The sample size was determined by the number of eligible patient records available during the study period.

### Data collection

Demographic variables included age, sex, and marital status. Clinical variables included cancer site, disease stage, metastatic status, and comorbidities (including diabetes mellitus, hypertension, dyslipidemia, NAFLD, thyroid dysfunction, and other chronic conditions). Biochemical parameters collected included serum 25(OH)D, and intact PTH.

### Biochemical measurements

Serum 25-hydroxyvitamin D [25(OH)D] levels were used to assess vitamin D status. Vitamin D deficiency was defined as <20 ng/mL, vitamin D insufficiency as 20–29 ng/mL, and vitamin D sufficiency as ≥30 ng/mL. Serum intact parathyroid hormone (iPTH) levels were measured using standard laboratory assays, with >65 pg/mL considered elevated based on laboratory reference ranges.

### Definition of functional vitamin D resistance

In accordance with previously published literature, functional vitamin D resistance was defined as the presence of elevated iPTH (>65 pg/mL) in the setting of sufficient serum 25(OH)D levels (≥30 ng/mL). (Lemke et al., 2021). Patients meeting these criteria were classified as having functional vitamin D resistance. Patients with sufficient vitamin D levels and normal or suppressed iPTH were classified as vitamin D–responsive.

### Outcomes

The primary outcome of this study was the prevalence of functional vitamin D resistance within a cohort of patients with solid and hematological malignancies. Secondary outcomes were exploratory and descriptive and included assessment of the relationship between serum 25(OH)D and iPTH concentrations as an indicator of vitamin D endocrine responsiveness, evaluation of the prevalence and distribution of vitamin D deficiency, insufficiency, and functional resistance across subgroups stratified by age, cancer type, disease stage, metastatic status, and metabolic comorbidities.

### Data analysis

Continuous variables were summarized as mean ± standard deviation, and categorical variables were expressed as frequencies and percentages. Group-wise comparisons of serum 25-hydroxyvitamin D and intact parathyroid hormone (iPTH) levels across age categories and cancer types were performed descriptively and visualized graphically. The association between serum 25-hydroxyvitamin D and iPTH concentrations was assessed using Pearson’s correlation coefficient. Multivariable binomial logistic regression analysis was employed to identify independent predictors of vitamin D deficiency, with results reported as odds ratios (ORs) and corresponding p values. Variables included in the model were selected *a priori* based on clinical relevance and included age, sex, tumor stage, metastatic status, and metabolic comorbidities. Due to the low prevalence of functional vitamin D resistance, regression modeling was not undertaken for this outcome; instead, associations with clinical and metabolic variables were evaluated using contingency analyses, with statistical significance assessed using appropriate categorical tests. A two-sided p value <0.05 was considered statistically significant.

## Results

### Baseline characteristics

A total of 220 patients with solid and hematological malignancies were included in the study (Table 1). The mean age was 57.6 ± 14.8 years, and the cohort was evenly distributed between males (49.5%) and females (50.9%). The most common cancer types were breast (18.2%), head and neck (13.6%), colorectal (11.8%), gynecological (11.8%), and upper gastrointestinal malignancies (9.1%). Most patients presented with advanced disease, with 57.7% having stage IV cancer and 49.5% having radiologically confirmed metastasis.

**TABLE 1:** Baseline demographic, clinical and biochemical characteristics of the study population (N = 220).

Characteristic	Value
Age (years), mean ± SD	57.6 ± 14.8
Sex, n (%)	
Male	109 (49.5)
Female	112 (50.9)
Marital status, n (%)	
Married	114 (51.8%)
Unmarried	106 (48.2%)
Cancer site, n (%)	
Breast	40 (18.2)
Cancer of unknown primary	1 (0.5)
Central nervous system	8 (3.6)
Colorectal	26 (11.8)
Genitourinary	18 (8.2)
Gynecological	26 (11.8)
Head and neck	30 (13.6)
Hematological malignancy	10 (4.5)
Hepatopancreatobiliary	19 (8.6)
Sarcoma/cutaneous	8 (3.6)
Thoracic	14 (6.4)
Upper gastrointestinal	20 (9.1)
Disease stage, n (%)	
Stage I	46 (20.9)
Stage II	38 (17.3)
Stage III	3 (1.4)
Stage IV	127 (57.7)
Metastasis, n (%)	
Present	109 (49.5)
Absent	111 (50.5)
Comorbidities, n (%)	
Type 2 diabetes mellitus	64 (29.1)
Dyslipidemia	7 (3.2)
Hypertension	59 (26.8)
NAFLD	2 (0.9)
Thyroid dysfunction	20 (9.1)
Other (CAD/CKD/autoimmune)	14 (6.4)
25-Hydroxyvitamin D (ng/mL), mean ± SD	22.2 ± 16.0
Intact PTH (pg/mL), mean ± SD	69.1 ± 66.3

Data are presented as mean ± SD, or n (%). Percentages may not sum to 100% due to missing data. NAFLD, non-alcoholic fatty liver disease; CAD, coronary artery disease; CKD, chronic kidney disease.

**FIGURE 1:**
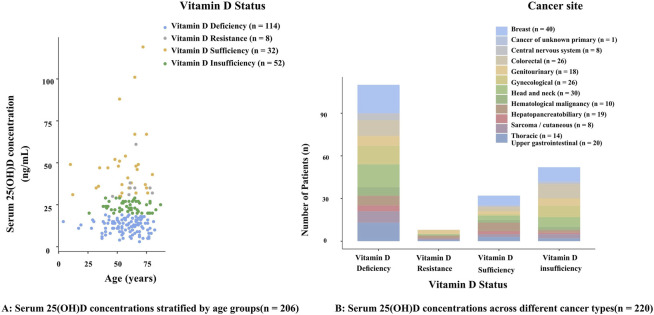
Distribution of serum 25-hydroxyvitamin D [25(OH)D] levels across age groups and cancer types. **(A)** Serum 25(OH)D concentrations stratified by age groups. **(B)** Serum 25(OH)D concentrations across different cancer types.

Metabolic comorbidities were common: 29.1% had type 2 diabetes mellitus and 26.8% had hypertension, while 3.2% had dyslipidemia. NAFLD was rare (0.9%), and thyroid dysfunction was present in 9.1% of patients. The mean serum 25-hydroxyvitamin D level was 22.2 ± 16.0 ng/mL, indicating a high prevalence of vitamin D insufficiency in the cohort. Mean intact parathyroid hormone (iPTH) level was 69.1 ± 66.3 pg/mL. Serum 25-hydroxyvitamin D levels varied widely across age groups and across different cancer types (Figures 1A,B). Serum 25-hydroxyvitamin D was significantly and inversely correlated with intact parathyroid hormone (iPTH) levels (Pearson’s r = −0.203, df = 189, p = 0.005), indicating that lower vitamin D concentrations were associated with higher PTH levels.

### Determinants of vitamin D deficiency

Multivariable binomial logistic regression was used to identify independent predictors of low vitamin D status (Table 2). Dyslipidemia was strongly associated with lower odds of low vitamin D status (OR 0.05, p = 0.009). Tumor burden was a major determinant; compared with stage I disease, patients with stage II cancer had markedly higher odds of low vitamin D status (OR 20.3, p = 0.004), and those with stage IV disease also showed significantly higher odds (OR 5.36, p = 0.037). Type 2 diabetes mellitus and hypertension were not independently associated with low vitamin D status. Some odds ratios, particularly for cancer stage and dyslipidemia, were large and should be interpreted with caution due to sparse data and limited event numbers. Regression analysis for functional vitamin D resistance was not performed due to low event rates.

**TABLE 2:** Multivariable binomial logistic regression for Vitamin D deficiency Outcome: Low Vitamin D status (Yes vs. No).

Predictor	β (SE)	Odds ratio	p value
Type 2 diabetes (Yes vs. no)	−0.18 (0.46)	0.84	0.700
Dyslipidemia (Yes vs. no)	−3.00 (1.14)	0.05	0.009
Hypertension (Yes vs. no)	−0.05 (0.46)	0.95	0.909
Stage II vs. stage I	3.01 (1.05)	20.3	0.004
Stage III vs. stage I	0.54 (1.45)	1.72	0.707
Stage IV vs. stage I	1.68 (0.81)	5.36	0.037

Model fit: AIC, 188; McFadden R^2^ = 0.127.

Estimates are regression coefficients (β) with standard errors (SE) and odds ratios. Stage I and absence of comorbidities were reference categories. Variables with extremely sparse data (e.g., NAFLD) were excluded. Analyses were performed using available-case data.

### Vitamin D resistance

Vitamin D resistance was uncommon, occurring in only 8 of 206 evaluable patients (3.9%). Because of this low event rate, regression modeling was not performed, and associations were examined using contingency analyses (Table 3). Due to the low number of events for functional vitamin D resistance (n = 8), multivariable regression analysis was not performed, and the findings are therefore based on descriptive and exploratory analyses.

**TABLE 3:** Association of metabolic and tumor variables with Vitamin D resistance.

Variable	Category	Vitamin D resistant n (%)	p value
Type 2 diabetes	No	2/144 (1.4%)	
	Yes	6/62 (9.7%)	0.010
Dyslipidemia	No	6/200 (3.0%)	
	Yes	2/6 (33.3%)	0.018
Hypertension	No	4/150 (2.7%)	
	Yes	4/56 (7.1%)	0.139
Cancer stage	I–IV	—	0.954
Metastasis	No	0/55 (0%)	
	Yes	8/150 (5.3%)	0.081

Vitamin D resistance was present in 8 of 206 patients (3.9%). Because of sparse cell counts, p values were derived using Fisher’s exact test.

Vitamin D resistance was strongly associated with metabolic disease. Resistance was significantly more frequent in patients with type 2 diabetes mellitus (9.7%) than in those without diabetes (1.4%; p = 0.010). Similarly, patients with dyslipidemia had a markedly higher prevalence of resistance (33.3%) compared with those without dyslipidemia (3.0%; p = 0.018). In contrast, vitamin D resistance was not significantly associated with tumor stage, metastasis, or hypertension.

## Discussion

In this study, we provide a comprehensive characterization of vitamin D status and functional vitamin D responsiveness in a heterogeneous cohort of patients with solid and hematological malignancies. Vitamin D insufficiency was highly prevalent, with mean serum 25-hydroxyvitamin D levels in the insufficient range and a significant inverse relationship between 25(OH)D and intact parathyroid hormone (iPTH). Beyond deficiency, we identified a small but clinically relevant subset of patients with functional vitamin D resistance, defined by elevated iPTH despite adequate circulating 25(OH)D. Notably, vitamin D deficiency was primarily associated with tumor burden, whereas functional vitamin D resistance clustered with metabolic comorbidities, particularly type 2 diabetes mellitus and dyslipidemia.

Consistent with prior reports, (Shaw et al., 2024; Lai et al., 2023), vitamin D deficiency and insufficiency were highly prevalent in this cohort, with a mean serum 25-hydroxyvitamin D concentration in the insufficient range. Our data also showed a significant inverse correlation between 25(OH)D and intact PTH as expected from normal feedback physiology. In addition to serum 25(OH)D levels, the measurement of intact parathyroid hormone (iPTH) may offer important clinical insights in oncology settings. While serum vitamin D concentrations are commonly used to assess deficiency, they may not fully reflect functional vitamin D activity at the tissue level. Elevated iPTH in the presence of adequate or borderline 25(OH)D levels may indicate a state of functional vitamin D resistance, suggesting impaired biological responsiveness. (Mukhopadhyay et al., 2019; Malik et al., 2020). Incorporating iPTH assessment alongside vitamin D measurements could therefore help identify patients who may not adequately respond to standard supplementation strategies. This approach may enable more individualized and physiologically informed vitamin D management, particularly in patients with cancer who often present with complex metabolic and inflammatory alterations.

Notably, advanced cancer stage was a major predictor of vitamin D deficiency in multivariable analysis, stages II and IV cancers had much higher odds of deficiency compared to stage I. This aligns with literature linking higher tumor burden to lower vitamin D status. (Wesa et al., 2015; Dev et al., 2011). This finding is consistent with the concept that advancing cancer is associated with systemic metabolic alterations, (Tufail et al., 2024), reduced nutritional intake, (Arends, 2024), altered hepatic metabolism, (Rosa-Caldwell et al., 2019), and chronic inflammation, (Kapoor et al., 2025), all of which may contribute to lower circulating vitamin D levels.

In the regression analysis, dyslipidemia was paradoxically associated with lower odds (0.05) of vitamin D deficiency. A potential explanation is that patients with dyslipidemia often receive statin therapy; statins have been reported to raise serum 25(OH)D levels modestly by inhibiting its catabolism via CYP3A4-mediated breakdown. (Cozma et al., 2025). The observed association between dyslipidemia and vitamin D status should be interpreted with appropriate caution. Given the retrospective and exploratory nature of the study, causality cannot be established. This relationship may reflect shared underlying mechanisms, including systemic inflammation, metabolic dysfunction, or alterations in lipid-mediated transport and bioavailability of vitamin D. However, an important limitation of this analysis is the absence of data on statin use and other lipid-lowering therapies, which may influence both lipid profiles and vitamin D metabolism. Consequently, the observed association cannot be fully contextualized, and the proposed explanations remain speculative.

Beyond deficiency, our study highlights the concept of functional vitamin D resistance. In our cohort, 8 out of 206 evaluable patients (3.9%) met this criterion. However, identification of resistance in this study should be interpreted with caution, as unmeasured confounding factors such as renal function, calcium intake, magnesium status, inflammatory markers, and active vitamin D supplementation may have contributed to the observed findings. The lack of these variables restricts a comprehensive interpretation of iPTH levels and limits the ability to accurately characterize functional vitamin D resistance. Nevertheless, these cases underscore that “normal” vitamin D levels do not always guarantee normal physiology. Further, its occurrence was significantly higher among patients with metabolic comorbidities, particularly type 2 diabetes mellitus and dyslipidemia. These associations are biologically plausible and align with prior evidence demonstrating that insulin resistance, lipid abnormalities, and chronic low-grade inflammation can impair vitamin D signaling through altered vitamin D receptor (VDR) expression, post-receptor signaling defects, and disrupted nuclear receptor function, leading to attenuated endocrine responses despite adequate circulating vitamin D levels. (Argano et al., 2023). Similar patterns of functional vitamin D resistance have been described in autoimmune diseases involving both chronic inflammation and metabolic dysfunction, where elevated PTH or impaired downstream signaling persists despite biochemical sufficiency. (Lemke et al., 2021).

Importantly, the occurrence of functional vitamin D resistance in patients with advanced or metastatic cancer, although based on limited numbers in the present study, raises the hypothesis that tumor burden, systemic inflammation, or cancer-related metabolic reprogramming may further exacerbate resistance to vitamin D signaling. This hypothesis warrants future prospective studies in larger, well-characterized cancer cohorts incorporating functional markers such as PTH, longitudinal assessment of disease progression, and detailed treatment stratification, to determine whether functional vitamin D resistance contributes to metastatic behavior or reflects a downstream consequence of advanced malignancy. Elucidating this relationship may have direct implications for individualized vitamin D supplementation strategies and therapeutic optimization in oncology.

### Limitations

There are several limitations in the present study. Being a retrospective study, causal relationships cannot be established. The low prevalence of functional vitamin D resistance in our cohort limits statistical power and restricts the ability to perform robust multivariable analyses. Consequently, these findings should be interpreted as exploratory and hypothesis-generating. As stated previously, important data, including calcium intake, renal function, inflammatory markers, vitamin D receptor activity, and active vitamin D metabolites, were not available. In addition, the effects of treatments such as vitamin D supplementation, chemotherapy, and corticosteroid use could not be fully assessed.

Despite these limitations, the study offers a nuanced view of vitamin D biology in cancer. While vitamin D deficiency was common, functional vitamin D resistance emerged as a distinct and relatively infrequent phenotype, more closely associated with metabolic comorbidity than with tumor characteristics alone. These findings support the concept that vitamin D deficiency and vitamin D resistance represent biologically distinct states with potentially different implications for supportive and integrative cancer care.

## Conclusion

Vitamin D deficiency and insufficiency were common across cancer types and stages, while functional vitamin D resistance, though less frequent, appeared as a distinct phenotype associated with metabolic comorbidities. These findings highlight heterogeneity in vitamin D responsiveness among patients with cancer and suggest that assessing vitamin D resistance may offer additional metabolic insight; however, these observations are exploratory and require validation in larger, controlled studies.

## Data Availability

The raw data supporting the conclusions of this article will be made available by the authors, without undue reservation.
